# Frailty Through a One Health Lens: Biological Sex, Mental Health, and Oral Function in Physically Active Older Adults

**DOI:** 10.3390/ijerph23040486

**Published:** 2026-04-12

**Authors:** Luciano Maia Alves Ferreira, José Brito, Catarina Colaço, Marcelo Palinkas, Ricardo Brites, Maia e Maia Fischel e Andrade, João Tiago Botelho, José João Baltazar Mendes, Selma Siessere, Simone Regalo

**Affiliations:** 1 Mathias Vitti Electromyography Laboratory, Faculty of Dentistry of Ribeirão Preto, University of São Paulo, São Paulo 05508-000, Brazil; lucianobatatais@hotmail.com (L.M.A.F.); selmas@forp.usp.br (S.S.); simone@forp.usp.br (S.R.); 2 Clinical Research Unit (CRU), Egas Moniz Center for Interdisciplinary Research (CiiEM), Egas Moniz School of Health & Science, 2829-511 Almada, Portugal; jbrito@egasmoniz.edu.pt (J.B.); ccolaco@egasmoniz.edu.pt (C.C.); jbotelho@egasmoniz.edu.pt (J.T.B.); jmendes@egasmoniz.edu.pt (J.J.B.M.); 3 Neuromodulation and Pain Laboratory (NEUROPAIN), Egas Moniz School of Health & Science, 2829-511 Almada, Portugal; ricardogbrites@gmail.com; 4 Laboratory of Neuroscience, Neuromodulation and Study of Pain (LANNED), Federal University of Alfenas (UNIFAL-MG), Alfenas 37130-001, Brazil; maiaemaiaandrade@gmail.com

**Keywords:** frailty, aging, health of the older adults, geriatric assessment, primary care

## Abstract

**Highlights:**

**Public health relevance—How does this work relate to a public health issue?**

- Frailty in older adults is a growing global public health challenge associated with disability, healthcare utilization, and mortality.
- This study explores frailty through a One Health perspective, integrating biological sex, mental health, and oral function in physically active older populations.

**Public health significance—Why is this work of significance to public health?**

- Identifies key determinants of frailty beyond physical inactivity, including sex differences and antidepressant use, highlighting vulnerable subgroups.
- Demonstrates the relevance of oral functional measures, such as bite force, as complementary indicators in geriatric assessment.

**Public health implications—What are the key implications or messages for practitioners, policy makers and/or researchers in public health?**

- Supports the integration of mental health, medication monitoring, and oral health evaluation into primary care strategies for aging populations.
- Reinforces the need for multidimensional and context-sensitive public health policies addressing frailty across different healthcare systems.

**Abstract:**

Frailty is a multifactorial geriatric syndrome marked by reduced physiological reserves and increased vulnerability to adverse outcomes. This multicenter observational study adopted a One Health approach to examine the association between frailty and biological sex, denture use, and antidepressant medication, as well as their impact on bite force, in two transnational cohorts of physically active older adults. The sample included 499 individuals aged ≥60 years (295 from Brazil and 204 from Portugal), all with functional dentition and regular physical activity. Frailty was assessed using the adapted Fried phenotype and classified as non-frail (G0), pre-frail (G1), or frail (G2). Oral health, depressive symptoms (CES-D), bite force, and self-reported use of dentures and antidepressants were analyzed. Frailty was significantly associated with biological sex (*p* < 0.001), with higher prevalence among women, especially in G2. Antidepressant use was associated with frailty in the Portuguese cohort (*p* < 0.001) and in the total sample (*p* = 0.005), but not in Brazil. No significant association was observed between denture use and frailty. However, Brazilian participants without dentures showed significantly higher bite force (*p* < 0.001), indicating a functional oral health effect. Frailty was associated with female sex and antidepressant use, while bite force emerged as a complementary functional marker for geriatric assessment.

## 1. Introduction

Frailty is a multifactorial geriatric syndrome characterized by reduced physiological reserves and impaired responsiveness to stressors, leading to increased vulnerability to adverse outcomes such as falls, hospitalization, loss of independence, and early mortality. Early identification is crucial for implementing interventions that can slow its progression and improve quality of life in older adults [1]. Among the available assessment models, the Fried phenotype is the most widely used, based on five clinical criteria: unintentional weight loss, self-reported exhaustion, low handgrip strength, slow gait, and reduced physical activity [2,3,4,5,6].

The prevalence of frailty increases with age and is shaped by biological, psychological, and social factors that reflect the dynamics of aging [2,3]. Biological sex is a relevant determinant: women generally have a higher prevalence across all age groups, likely due to differences in hormonal levels, body composition, and chronic disease burden. On the other hand, frail men face a higher risk of mortality, suggesting a more severe expression of the phenotype in this group [7,8,9,10].

In addition to biological characteristics, functional aspects such as oral health are increasingly recognized as essential in geriatric assessment. Tooth loss, reduced bite force, and inadequate denture use are associated with inadequate dietary intake, sarcopenia, chronic inflammation, and functional decline, all of which can exacerbate frailty [10,11]. Efficient chewing supports nutrition, cognition, and autonomy, while properly fitted dentures can partially compensate for functional deficits, depending on their type, fit, and duration of use [12,13].

Bite force has emerged as a relevant functional marker for the early detection of frailty, as it reflects the condition of the orofacial muscles and, indirectly, overall functional integrity [14]. Bite force correlates with nutritional capacity, oral health, and physical performance and can serve as a noninvasive biomarker for clinical monitoring [15,16,17,18].

Mental health also plays a crucial role. Depression, highly prevalent in older adults, has a bidirectional relationship with frailty: frail individuals are more likely to develop depressive symptoms, while depression can accelerate frailty through reduced physical activity, poor nutrition, and lower treatment adherence [19,20]. Furthermore, the use of antidepressants, although clinically important, can have adverse effects on balance, cognition, and functionality, thus worsening frailty outcomes [21].

Adopting a One Health perspective is particularly relevant for studying frailty in older adults, as it emphasizes the integration of biological, psychological, and functional determinants of health within social and environmental contexts. Frailty is influenced not only by biomedical factors but also by mental health, medication use, and oral functionality, which interact with cultural and systemic conditions. This multidimensional approach aligns with the United Nations’ Sustainable Development Goals, particularly SDG 3 (Good Health and Well-Being) and SDG 10 (Reduced Inequalities), by promoting equitable access to healthcare, preventive strategies, and interventions that support healthy and autonomous aging [22]. By incorporating these dimensions, the study integrates scientific, clinical, and social perspectives, providing a framework for comprehensive frailty assessment and context-sensitive interventions in community-dwelling older adults across different countries.

Comparative studies across populations help elucidate how cultural, social, and economic contexts influence frailty and their associations with gender, oral health, and medication use [23]. Investigating older adults in countries with distinct demographic and health systems, such as Brazil and Portugal, can provide valuable insights for personalized prevention and treatment strategies.

In this study, the One Health perspective is operationalized by examining the interdependence between biological sex, mental health pharmacological management, and oral functional status. This holistic framework moves beyond traditional geriatric assessments by viewing frailty as a syndemic outcome of internal biological environments and external healthcare access contexts [22]. In this regard, the Brazilian Unified Health System (SUS) and the Portuguese National Health Service (SNS) provide distinct structural backgrounds for oral and mental health care, which may influence frailty trajectories in physically active older adults.

In this perspective, the One Health approach is operationalized by recognizing oral health and mental health as interdependent components of an integrated human system. Evidence suggests that oral conditions (e.g., tooth loss, reduced bite force) and psychological status (e.g., depressive symptoms) are both influenced by biological processes and socio-environmental determinants and jointly impact functional capacity and aging trajectories [24]. This integrated view supports the concept that systemic balance in older adults depends on the interaction between physiological and psychosocial domains.

This study examines the association between frailty and qualitative variables, including gender, denture use, and antidepressant medication, among physically active older adults in Brazil and Portugal. By excluding low physical activity levels, we specifically focused on other determinants of frailty and their impact on bite force as a functional marker of oral health. The originality of this work lies in integrating biological, psychological, and oral health dimensions within a cross-national framework, highlighting how sociocultural and health contexts shape frailty. This multidimensional approach provides new evidence and points to potential clinical indicators for screening and managing frailty in primary care.

## 2. Materials and Methods

### 2.1. Ethical Aspects

The study was approved by the Research Ethics Committee of the University of São Paulo, Ribeirão Preto School of Dentistry (process # 79222024.8.0000.5419, on 25 April 2024). All participants were informed about the objectives of the study and signed an Informed Consent Form (ICF). Participation was voluntary, with a guarantee of confidentiality and freedom to withdraw at any time, without losses or costs.

This study is a cross-sectional analysis of baseline data from a larger multicenter longitudinal project conducted in Brazil and Portugal. The sample consisted of 499 community-dwelling senior people aged 60 years or older practicing regular physical activity. The present analysis aimed to verify the association between frailty and qualitative variables of interest, such as biological sex, use of dental prostheses and use of antidepressant medication.

### 2.2. Sample

Initially, 550 older adults were identified, of which 499 were included in the final analysis, 296 Brazilians (CASI project—Center for Support and Health of the Seniors, Batatais/SP) and 204 Portuguese (project “Sempre a Mexer”, Sesimbra, Setúbal). All of them performed at least 150 min of physical activity per week, according to the International Physical Activity Questionnaire (IPAQ).

### 2.3. Eligibility Criteria

The inclusion criteria were for individuals aged 60 years or older, with preserved cognition, functional autonomy, at least 20 natural or functionally rehabilitated teeth (with fixed or removable prosthesis), and participation in regular physical activity programs. Exclusion criteria were defined for individuals who were completely edentulous, with more than 20 missing teeth without rehabilitation, or with a diagnosis of degenerative neurological pathologies that compromised cognitive function.

### 2.4. Frailty Assessment

The frailty phenotype was adapted from Fried’s criteria [1] to include objective functional markers suitable for a physically active population. Five criteria were used: unintentional weight loss, self-reported exhaustion, low physical activity (IPAQ), slow gait speed (TUG test), and muscular weakness (assessed by bite force and handgrip strength). Participants were classified as non-frail (0 criteria), pre-frail (1–2 criteria), or frail (≥3 criteria).

### 2.5. Data Evaluation

Data collection involved standardized instruments for physical, functional, and subjective health assessment. Data was collected by a team of previously trained researchers to ensure inter-examiner reliability. Frailty was identified using a method adapted from the phenotype proposed by Fried [2], incorporating variables such as body composition, handgrip strength, functional performance tests (Timed Up and Go and Sit-to-Stand), and bite force. Participants were classified into three categories: G0 (non-frail: 0 positive criteria), G1 (pre-frail: 1–2 positive criteria), and G2 (frail: 3 or more positive criteria). Additionally, qualitative variables included: depression (Center for Epidemiologic Studies Depression Scale—CES-D), use of dental prostheses, and use of antidepressants. Body composition was assessed using a Bioimpedance analyzer (Accuniq BC300, Selvas Healthcare, Daejeon, Korea). Maximum bite force was measured with a digital bite dynamometer (EMG System do Brazil, São José dos Campos, Brazil), while handgrip and forceps grip strength were evaluated using KINVENT dynamometers (KINVENT, Montpellier, France). All procedures followed the manufacturers’ instructions to ensure measurement precision.

### 2.6. Statistical Analysis

Associations between fragility and categorical variables such as sex, use of dental prosthetics, and antidepressive medication were evaluated using chi-square tests of independence. Because these tests were performed both in the full sample and separately within two cohorts, a correction for multiple comparisons was applied to control the family-wise type I error rate at 5%. As nine independence tests were conducted, the significance threshold was adjusted to 0.005, providing a conservative criterion for statistical significance.

Bite force was analyzed using two-way analyses of variance (ANOVA), conducted separately for the Portuguese and Brazilian cohorts. Each model included two fixed factors—use of dental prosthesis (yes/no) and use of antidepressant medication (yes/no)—as well as their interaction. Type III sums of squares were used in all analyses.

Assumptions for the two-way ANOVA were evaluated prior to model estimation in the cells defined by the combinations of the two fixed factors. These checks included assessing the normality of the dependent variable within each cell using the Shapiro–Wilk test and evaluating homogeneity of variances across cells using Levene’s test.

In the Portuguese cohort, non-normal distributions were detected in two cells, one of which showed only moderate skewness. Variances were heterogeneous across cells, and cell sizes were unequal (*n* = 24–95). Because these conditions may affect the validity of the classical ANOVA, two assumption-free factorial methods were applied for verification: a robust two-way ANOVA based on trimmed means (WRS2) and the Aligned Rank Transform procedure (ARTool). These analyses were used exclusively in the Portuguese cohort to assess the robustness of the classical ANOVA results.

In the Brazilian cohort, the distribution of bite force was non-normal but symmetric in all cells, after removal of a single outlier. Given the large cell sizes, ANOVA is considered robust to deviations from normality under these conditions. In addition, bite force showed homogeneous variances across cells, further supporting the appropriateness of ANOVA for this cohort. Therefore, for consistency across cohorts, the classical ANOVA was applied and its results reported for both datasets, with nonparametric analyses used only as a robustness check in the Portuguese cohort.

Effect sizes were reported using partial eta squared (η^2^_p_) to quantify the magnitude of group differences. For a clearer assessment of the practical significance of the observed effects, η^2^_p_ values were also converted to Cohen’s f, which expresses effect size on a standardized scale commonly used in ANOVA. According to convention, Cohen’s f values of approximately 0.10, 0.25, and 0.40 correspond to small, medium, and large effects, respectively. Observed power values are presented descriptively to indicate the sensitivity of the analyses, but they were not used as inferential criteria. Given the cross-sectional design, the analyses assess group differences and factor effects without implying causality.

All statistical tests were conducted at the 5% significance level using IBM SPSS Statistics 30, except for the nonparametric analyses in the Portuguese cohort, which were performed with the WRS2 and ARTool packages (Version 0.11.2) in R.

## 3. Results

The final sample of the study was composed of 499 physically active older adults, 295 of whom lived in Brazil and 204 in Portugal. All participants met the inclusion criteria, including age 60 years or older, functional dentition, and regular physical activity. The initial descriptive analysis revealed significant differences between the groups in terms of sociodemographic, anthropometric and functional variables. Next, comparative and association analyses were performed between the different domains evaluated, including muscle strength (manual and bite), balance, mobility, oral health, depressive symptoms, and level of physical activity, with the aim of identifying the main predictors of frailty status in the older adults in the sample. Table 1 presents the mean, standard deviation (SD), minimum (Min) and maximum (Max) values for each variable in the samples of Brazil and Portugal.

Table 2 identifies the association between gender and frailty. The chi-square test of independence between gender and frailty, followed by paired comparisons of the proportion of male/female individuals for each frailty subgroup, detected a significant association between the two variables according to the following results:

- Brazil cohort: χ^2^ = 23.531, df = 2, sig < 0.001; the proportion of sex = 1 (M) in the frailty groups G0 and G1 is higher than that of sex = 0 (F), while the proportion of sex = 1 (M) in the frailty group G2 is lower than that of sex = 0 (F).
- Portugal cohort: χ^2^ = 29.654, df = 2, sig < 0.001; the proportion of sex = 1 (M) in the frailty group G0 is greater than that of sex = 0 (F), and it is equal to that of sex = 0 (F) in the frailty group G1, while the proportion of sex = 1 (M) in the frailty group G2 is lower than that of sex = 0 (F).
- Integer group: χ^2^ = 43.363, df = 2, sig < 0.001; the proportion of sex = 1 (M) in the frailty group G0 is greater than that of sex = 0 (F), and it is equal to that of sex = 0 (F) in the frailty group G1, while the proportion of sex = 1 (M) in the frailty group G2 is lower than that of sex = 0 (F).

Table 3 shows the association between the use of dental prostheses and frailty. The chi-square test for independence between the use of dental prostheses and frailty did not detect a significant association in Brazil (χ^2^ = 1.069, df = 2, sig < 0.586), Portugal (χ^2^ = 1.105, df = 2, sig < 0.575), or the total group (χ^2^ = 4.331, df = 2, sig < 0.115).

Table 4 shows the association between the use of antidepressant medication and frailty. The chi-square test of independence between the use of antidepressant medication and frailty, followed by paired comparisons of the proportion of males and females for each frailty subgroup, produced the following results:

- Brazil cohort: χ^2^ = 0.173, df = 2, sig = 0.917, which shows a non-significant association between the attributes.
- Portugal cohort: χ^2^ = 14.247, df = 2, sig < 0.001, which shows a significant association between the variables; the proportion of antidepressants = 0 (No) in frailty group G0 is higher than that of antidepressants = 1 (Yes), it is equal to that of antidepressants = 1 (Yes) in frailty group G1, while the proportion of antidepressants = 0 (No) in frailty group G2 is lower than that of antidepressants = 1 (Yes).
- Whole group: χ^2^ = 10.806, df = 2, sig = 0.005, showing a significant association between the attributes, with the same distribution of proportions observed in the Portugal cohort.

Table 5 shows the effect of the use of antidepressant medication and dental prostheses on bite force.

In the Portuguese cohort (Table 5), the two-way ANOVA indicated no significant main effect of dental prosthesis use, F(1.200) = 0.481, *p* = 0.489, with a very small effect size (η^2^_p_ = 0.002; Cohen’s f = 0.05). Likewise, there was no significant main effect of antidepressant use, F(1.200) = 0.389, *p* = 0.534, also with a very small effect size (η^2^_p_ = 0.002; Cohen’s f = 0.05). The interaction between the two factors approached significance, F(1.200) = 3.817, *p* = 0.052, with a small effect size (η^2^_p_ = 0.019; Cohen’s f = 0.14). The model accounted for a modest proportion of variance (R^2^ = 0.036).

Because normality and homogeneity assumptions were violated in this cohort, the results were verified using nonparametric factorial methods. Both the WRS2 robust ANOVA and the ARTool analysis reproduced the pattern observed in the classical ANOVA, showing no significant main effects or interaction. This convergence indicates that the observed group differences are stable across methods and not driven by assumption violations.

In the Brazilian cohort (Table 5), the ANOVA revealed a significant main effect of dental prosthesis use on bite strength, F(1.290) = 11.088, *p* < 0.001, with a small-to-medium effect size (η^2^_p_ = 0.037; Cohen’s f = 0.20). Although the effect size was small, the observed power for this effect was high (0.913), indicating that the analysis had sufficient sensitivity to detect group differences of this magnitude and reducing the likelihood that the result reflects sampling variability. No significant effect of antidepressant use was observed, F(1.290) = 0.133, *p* = 0.716, with a negligible effect size (η^2^_p_ ≈ 0.000; Cohen’s f ≈ 0.00). The interaction between factors was also not significant, F(1.290) = 0.655, *p* = 0.419, with a very small effect size (η^2^_p_ = 0.002; Cohen’s f = 0.05). The model explained a small proportion of variance (R^2^ = 0.037). It is noted that these effects did not change with the inclusion of the outlier observation in the data analysis.

Because the Brazilian cohort met the assumptions of symmetry and homoscedasticity, and sample sizes were large, the small but statistically reliable difference between prosthesis groups is unlikely to be attributable to assumption violations.

**Figure 1:**
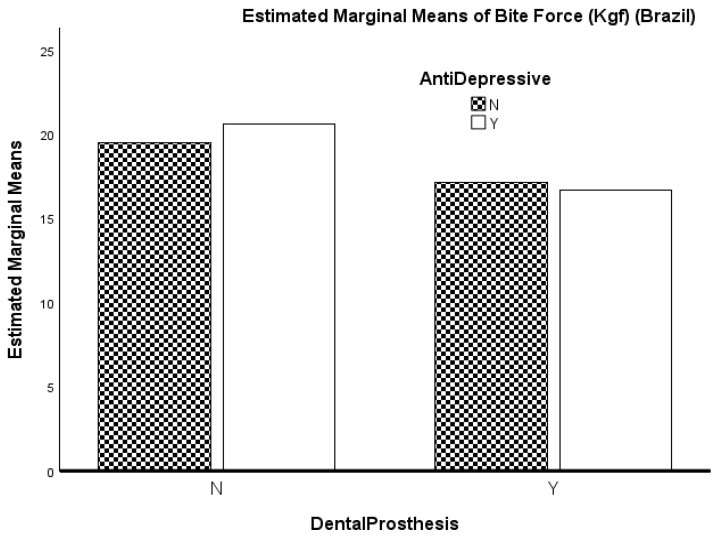
Marginal means of bite force (Brazil).

**Figure 2:**
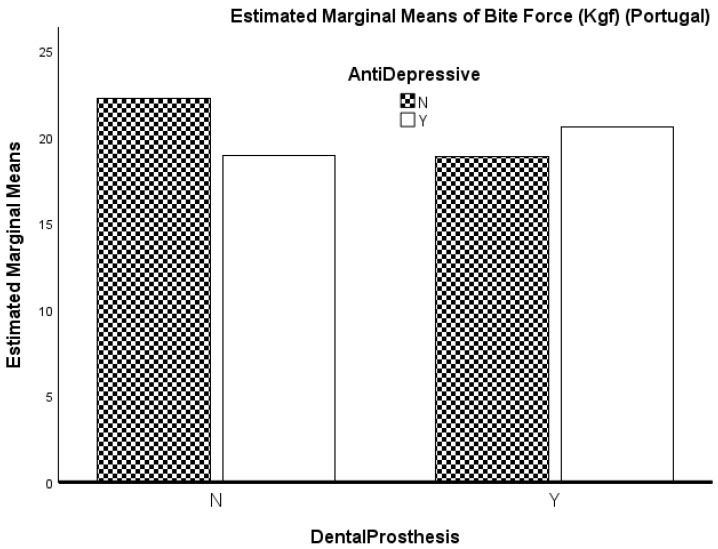
Marginal means of bite force (Portugal).

These results are observed for the whole group, with the effect of the use of dental prostheses reaching statistical significance (*p* < 0.001) and no other relevant results (Figure 1 and Figure 2).

Although the estimated marginal means in Figure 2 suggest a possible crossover pattern between dental prosthesis use and antidepressant medication in the Portuguese cohort, this pattern was not supported by the inferential model. As mentioned above, the Dental Prosthesis × antidepressant interaction did not reach statistical significance, indicating that the apparent difference in the figure should be interpreted as descriptive rather than as evidence of a reliable interaction effect.

## 4. Discussion

This study suggests that frailty in physically active older adults is significantly associated with qualitative factors, such as biological sex and antidepressant use, with marked differences between the Brazilian and Portuguese cohorts. In contrast, the use of dentures was not statistically associated with frailty, although it appeared to influence bite force, particularly in Brazil.

The findings regarding sex align with previous evidence of a higher prevalence of frailty in women, even among physically active older adults [25,26]. This disparity may be explained by lower muscle mass, a higher prevalence of osteoarticular diseases, and inequalities in access to healthcare throughout life. Furthermore, women, although more frail, tend to live longer, perpetuating the “gender paradox” of aging [27]. Conversely, the lower proportion of frail men may mask a higher mortality risk with the development of frailty, given its more severe expression in this group.

Depression, often underdiagnosed in older adults, emerged as a relevant factor associated with frailty, particularly in Portugal. This aligns with evidence supporting a potential bidirectional relationship between these conditions [28,29]. Depressive symptoms reduce physical activity, treatment adherence, and social participation, contributing to cognitive impairment and isolation, factors that intensify the risk of frailty. Furthermore, certain antidepressants, such as tricyclics, could potentially impair cognition and balance, increasing the likelihood of functional decline [30]. These findings emphasize the need for integrated mental health approaches in geriatric care, with careful prescribing of psychotropic medications.

The prevalence of frailty and pre-frailty was higher in the Brazilian cohort than in the Portuguese cohort, but the association between frailty and antidepressant use was significant only in Portugal. This observation may reflect structural differences in health systems. In Brazil, despite universal access through the SUS, regional disparities and social inequalities might influence the expression of frailty [31]. In Portugal, although oral health care in the SNS is more restricted, greater integration of mental health with primary care may explain the significant link between antidepressant use and frailty, despite the lower overall prevalence [10,31].

However, these cross-country differences must be interpreted with caution. A significant limitation of the present study is the lack of control for socioeconomic status (SES) and educational level. In both cohorts, SES is a known determinant of health literacy and access to advanced dental rehabilitation, which directly impacts bite force and nutritional status. Therefore, the structural explanations provided here remain exploratory.

In the Portuguese cohort, the graphical representation of estimated marginal means (Figure 2) suggested a possible crossover pattern between dental prosthesis use and antidepressant medication. However, this pattern did not reach statistical significance in the corresponding two-way ANOVA. Accordingly, this apparent interaction should be regarded as descriptive rather than as evidence of a reliable effect and is best interpreted as hypothesis-generating.

Although the use of dentures has not been directly associated with frailty, cohort-specific differences in bite force may reflect healthcare structures. In Brazil, older adults without dentures have higher bite forces, suggesting that large-scale oral rehabilitation strategies, despite SUS initiatives, may not always provide well-fitting dentures capable of fully restoring masticatory function [30]. In Portugal, where dentures are less common but often obtained privately, they tend to be better fitted, restoring efficiency. Thus, while a direct relationship between denture use and frailty was not established, the present findings highlight the potential role of oral health systems in shaping functional performance, with downstream effects on nutrition and potentially on frailty trajectories [10,32,33]. Within a One Health perspective, these results reinforce the relevance of integrating oral and systemic health domains. In this context, recent evidence, including Ferreira et al. [18], supports bite force as an emerging functional biomarker in older adults and suggests its potential role as an independent indicator of frailty [17,34,35].

It is important to note that this study assessed the presence of dental prostheses but did not evaluate clinical parameters such as ‘denture fit’, stability, or the age of the prosthesis. A poorly adapted prosthesis can lead to functional limitations not captured by the mere classification of ‘denture user’. Future research should include the Oral Health Impact Profile (OHIP) or clinical quality indices to further clarify this relationship.

The lack of a direct association between denture use and frailty may be partially explained by the inclusion criterion requiring functional dentition, which likely reduced the impact of oral health on outcomes. However, ANOVA results indicated that older adults without dentures had greater bite force, reinforcing its potential value as a functional indicator in the multidimensional assessment of frailty.

A One Health perspective highlights that frailty results from the interplay of biological, psychological, and functional factors within specific contexts. The associations observed between frailty, female sex, antidepressant use, and bite force underscore the potential need for multidimensional assessment, integrating mental health and oral functionality. This approach also emphasizes how cultural and systemic differences between Brazil and Portugal may shape frailty expression and inform context-sensitive prevention strategies.

An important methodological limitation is the use of convenience sampling, which may introduce selection bias and limit the representativeness of the sample. As participants were recruited from specific community programs involving physically active older adults, the findings may not be generalizable to the broader older population, particularly those with lower functional status or reduced access to health services.

## 5. Limitations

This study has several limitations. First, its cross-sectional design precludes causal inferences, restricting interpretation to associations. Second, the use of antidepressants was considered as a proxy for mental health management and does not necessarily reflect the severity or duration of depressive symptoms. Third, the study did not assess important clinical variables related to oral rehabilitation, such as denture fit, stability, or duration of use, which may influence bite force outcomes. Fourth, potential confounding variables, including socioeconomic status and educational level, were not controlled for, which may affect both oral health and frailty. Fifth, the use of convenience sampling introduces potential selection bias and limits the external validity and generalizability of the findings. Finally, the sample consisted exclusively of physically active older adults, which may reflect a “healthy participant effect” and reduce applicability to more sedentary or institutionalized populations.

## 6. Conclusions

Frailty in physically active older adults is strongly associated with female sex and antidepressant use, with these relationships particularly evident in the Portuguese cohort, highlighting the influence of cultural and healthcare contexts. Although denture use was not directly linked to frailty, its impact on bite force underscores the relevance of functional oral health as a complementary marker in geriatric assessment.

A One Health perspective emphasizes that frailty emerges from the interplay of biological, psychological, and functional factors within specific social and systemic contexts. These findings support the integration of mental health evaluation, medication monitoring, and functional oral assessments into primary care screening, promoting early identification and multidimensional management of frailty. Future longitudinal and multicenter studies are warranted to clarify causal pathways, evaluate long-term outcomes, and develop context-sensitive strategies that foster healthier, more autonomous aging.

Within a One Health framework, bite force may represent a promising, low-cost functional indicator associated with oral function and overall physiological status. Previous studies have suggested its potential role as an independent marker of frailty; however, this relationship was not directly examined in the present study and should be confirmed in future research.

## Figures and Tables

**Table 1:** Descriptive statistics.

Region	Variable	*n*	Min.	Max.	Average	SD
**Brazil**	Age (years)	295	55.0	92.0	72.6	6.5
	Right grip strength (Kgf)	295	4.5	43.9	17.1	6.0
	CES-D	295	0.0	42.0	12.9	9.1
	TUG (seconds)	295	5.0	27.4	10.3	2.9
	OFI-8/EN	295	1.0	11.0	4.5	2.2
	Body Fat (%)	295	3.0	60.0	36.7	11.0
	STS (repeats)	295	4.0	24.0	11.1	3.3
	Left grip strength (Kgf)	295	5.8	39.7	16.7	6.0
	Bite force (Kgf)	295	2.0	61.2	18.0	7.7
	BMI (kg/m^2^)	295	14.6	44.0	28.4	5.3
	WHR (waist-to-hip ratio)	295	0.4	1.1	0.9	0.1
**Portugal**	Age (years)	204	56.0	92.0	73.7	6.6
	Right grip strength (Kgf)	204	6.8	40.2	16.6	5.0
	CES-D	204	0.0	42.0	13.4	9.1
	TUG (seconds)	204	4.4	17.8	7.6	1.9
	OFI-8/EN	204	1.0	10.0	4.5	2.0
	Body Fat (%)	204	7.0	52.1	34.5	6.4
	STS (repeats)	204	6.0	30.0	16.3	3.8
	Left grip strength (Kgf)	204	5.1	33.8	16.2	4.8
	Bite force (Kgf)	204	2.9	35.4	20.1	7.9
	BMI (kg/m^2^)	204	18.2	45.6	27.4	4.4
	WHR (waist-to-hip ratio)	204	0.7	1.2	0.9	0.1

Abbreviations: BMI, Body Mass Index; CES-D, Center for Epidemiologic Studies Depression Scale; G0, Non-frail; G1, Pre-frail; G2, Frail; OFI-8/EN, Oral Function Inventory; SD, Standard Deviation; STS, Sit-to-Stand test; TUG, Timed Up and Go test. Note: Based on this data, the classifications between G0, G1 and G2 were made.

**Table 2:** Frailty versus Sex.

Cohort					
Region			Sex		Total
			0 (F)	1 (M)	
**Brazil**	**Frailty**	G0	22	13	35
		G1	95	38	133
		G2	117	10	127
	**Total**		234	61	295
**Portugal**	**Frailty**	G0	49	28	77
		G1	62	8	70
		G2	56	1	57
	**Total**		167	37	204

**Table 3:** Frailty versus use of dental prostheses (Crosstabulation).

Count					
Region			Wear Dentures		Total
			1 (Yes)	0 (No)	
**Brazil**	**Frailty**	G0	21	14	35
		G1	92	41	133
		G2	86	41	127
	**Total**		199	96	295
**Portugal**	**Frailty**	G0	44	33	77
		G1	45	25	70
		G2	32	25	57
	**Total**		121	83	204

**Table 4:** Frailty versus antidepressant medication.

Cohort					
Region			Antidepressant Medication		Total
			1 (Yes)	0 (No)	
**Brazil**	**Frailty**	G0	13	22	35
		G1	48	85	133
		G2	49	78	127
	**Total**		110	185	295
**Portugal**	**Frailty**	G0	11	66	77
		G1	15	55	70
		G2	24	33	57
	**Total**		50	154	204

**Table 5:** (**A**). Tests of between-subjects effects—Portugal (DV: Bite Strength, Kgf). (**B**). Tests of between-subjects effects—Brazil (DV: Bite Strength, Kgf; one outlier removed).

**A**								
	**SS**	**df**	**MS**	**F**	**Sig.**	**η^2^_p_**	**f**	**Power**
Corrected Model	457.396	3	152.465	2.494	0.061	0.036	-	0.612
Intercept	60,417.431	1	60,417.431	988.134	<0.001	0.832	-	1.000
DentalProsthesis	29.435	1	29.435	0.481	0.489	0.002	0.050	0.106
AntiDepressive	23.782	1	23.782	0.389	0.534	0.002	0.050	0.095
DentalProsthesis × AntiDepressive	233.386	1	233.386	3.817	0.052	0.019	0.140	0.494
Error	12,228.591	200	61.143	-	-	-	-	-
**B**								
	**SS**	**df**	**MS**	**F**	**Sig.**	**η^2^_p_**	**f**	**Power**
Corrected Model	582.013	3	194.004	3.703	0.012	0.037	-	0.802
Intercept	80,554.784	1	80,554.784	1537.533	<0.001	0.841	-	1.000
DentalProsthesis	580.900	1	580.900	11.088	0.001	0.037	0.200	0.913
AntiDepressive	6.972	1	6.972	0.133	0.716	0.000	0.000	0.065
DentalProsthesis × AntiDepressive	34.330	1	34.330	0.655	0.419	0.002	0.050	0.127
Error	15,193.749	290	52.392	-	-	-	-	-

A: R^2^ = 0.036 (Adjusted R^2^ = 0.022); α = 0.05. B: R^2^ = 0.037 (Adjusted R^2^ = 0.027); α = 0.05.

## Data Availability

The data used in this study are available from the corresponding author upon reasonable requests.
